# A functional type I topoisomerase from *Pseudomonas aeruginosa*

**DOI:** 10.1186/1471-2199-10-23

**Published:** 2009-03-24

**Authors:** Teesta Jain, Benjamin J Roper, Anne Grove

**Affiliations:** 1 Department of Biological Sciences, Louisiana State University, Baton Rouge, LA 70803, USA; 2 University of Texas Medical Branch, Galveston, TX, USA

## Abstract

**Background:**

*Pseudomonas aeruginosa *encodes a putative topoisomerase with sequence similarity to the eukaryotic type IB topoisomerase from Vaccinia virus. Residues in the active site are conserved, notably Tyr292 which would be predicted to form the transient covalent bond to DNA.

**Results:**

The gene encoding the *P. aeruginosa *topoisomerase I was cloned and expressed in *E. coli*. The enzyme relaxes supercoiled DNA, while a mutant containing a Tyr292 to Phe substitution at the active site was found to be catalytically inert. This is consistent with the role of Tyr in forming the covalent intermediate. Like Vaccinia topoisomerase, the *P. aeruginosa topoisomerase *relaxes DNA in the absence of ATP, but unlike Vaccinia topoisomerase, *P. aeruginosa *topoisomerase does not relax supercoiled DNA without MgCl_2 _present. In addition, high concentration of NaCl is not able to substitute for MgCl_2 _as seen for Vaccinia topoisomerase. A truncated derivative of the topoisomerase lacking residues 1–98 relaxes DNA, with both full length and truncated enzyme exhibiting equivalent requirements for divalent cations and the ability to relax DNA to completion, suggesting a shared domain organization. DNA-binding assays suggest an only modest preference for the CCCTT pentameric sequence required for transesterification by Vaccinia topoisomerase IB.

**Conclusion:**

*P. aeruginosa *encodes a functional topoisomerase with significant similarity to the type IB enzyme encoded by poxviruses. In contrast to the Vaccinia-encoded homolog, the *P. aeruginosa*-encoded enzyme requires divalent cations for catalytic activity, relaxes DNA to completion, and does not exhibit a strong preference for the pentameric sequence stringently required by the Vaccinia-encoded homolog. A comparison with the structure of poxviral topoisomerase in complex with DNA suggests that bacterial homologs of the eukaryotic type IB topoisomerase may exhibit a relaxed sequence preference due to the lack of conservation of certain residues involved in sequence-specific DNA contacts, and that interaction with an only modestly preferred sequence may result in suboptimal positioning of catalytic residues.

## Background

DNA topoisomerases are enzymes that solve topological problems associated with important processes such as DNA replication, transcription, recombination and chromatin remodeling by introducing transient single or double stranded breaks in the DNA and releasing accumulated strain [1]. DNA cleavage leads to a transient phosphodiester bond between a tyrosine at the active site of the protein and one of the broken DNA strands. The final step in the reaction is resealing of the DNA break, the end result being an altered DNA linking number [1]. DNA topoisomerases are of two kinds; those enzymes that cleave only one DNA strand are type I topoisomerases and those enzymes that cleave both DNA strands are referred to as type II. The type I topoisomerases are further classified as type IA or IB. In the type IA subfamily, the protein is linked to the 5' phosphate and in the type IB subfamily, the protein is linked to the 3' phosphate of the DNA substrate [1]. The topoisomerases have attracted attention precisely because of their diverse roles in controlling DNA topology and therefore in regulating DNA-dependent processes. They can act as potential targets for anticancer drugs; for example, the plant alkaloid camptothecin blocks DNA and RNA synthesis in the treated cells and acts as an anticancer drug for human cancer by targeting the topoisomerase I, causing accumulation of lethal DNA strand breaks [2]. Coumermycin, which targets the Vaccinia topoisomerase, interferes with enzyme-DNA complex formation [3]. Both the DNA topoisomerases and the site-specific DNA recombinases perform cleavage and ligation reactions [1,2,4-11]. Therefore, the topoisomerases can also provide insight to the function of their close cousins, the tyrosine recombinases, which could also serve as drug targets.

Besides the linkage of the protein to either the 3'- or 5'- end of the DNA, there is another difference in catalytic mechanism between the type IA and type IB topoisomerases. In reactions catalysed by the type IA enzymes, the breaking of the duplex DNA and the rejoining event occurring after the stress relief occurs in a single stranded region of the DNA template [7,12,13]. This is in contrast with the type IB topoisomerases, which generate a nick in a double stranded region of the DNA template [1,7,14,15]. The type IB topoisomerase might be able to attack a single stranded region in a double stranded DNA template but when such a reaction occurs the enzyme might detach from the 5' end and yield a linear intermediate [1,16].

In contrast to the type I topoisomerases, the type II topoisomerases catalyze the breakage of both strands of DNA and also catalyze the catenation and decatenation of double stranded DNA circles [17-19]. The type II enzymes use the energy released from ATP hydrolysis to create breaks in the double stranded DNA, to cause the nearby double helix to pass through this beak, to reseal the break and dissociate the DNA [7]. By contrast, the type I enzymes do not require ATP.

The eukaryotic type IB DNA topoisomerase family includes the topoisomerase I encoded by the Vaccinia virus [20,21]. It is the smallest topoisomerase known, a 314 amino acid protein that binds duplex DNA with stringent specificity for transesterification at 5'-(C/T)CCTT sites [15,21]. Indeed, all poxviruses studied encode a topoisomerase IB homolog [22-26]. The Vaccinia virus DNA topoisomerase I is biochemically similar to the eukaryotic type I topoisomerase and yet is only one-third the size. Proteins with homology to the Vaccinia topoisomerase IB are also encoded by a few eubacteria; for example, the *Pseudomonas aeruginosa *topoisomerase I (PAT) shares significant sequence similarity with Vaccinia virus topoisomerase IB. The sizes of the bacterial enzymes are similar to that of poxviral enzymes, and the tyrosine nucleophiles as well as other residues implicated in catalysis are well conserved. Therefore, the *P. aeruginosa *topoisomerase I can act as a model to study the mechanism of topoisomerases as well as provide important insight into the physiology of *P. aeruginosa*. A functional eubacterial topoisomerase IB has so far only been described in *Deinococcus radiodurans *[27,28].

*P. aeruginosa *is a bacterium responsible for severe nosocomial infections, life-threatening infections in immunocompromised persons, and chronic infections in cystic fibrosis patients. The bacterium's virulence depends on a large number of cell-associated and extracellular factors. Considering that topoisomerases serve vital cellular functions, the topoisomerase from this organism may be a potential drug target as well [29]. In the present study, we show that *P. aeruginosa *encodes a functional topoisomerase I (PAT) with sequence similarity to the Vaccinia virus and *D. radiodurans *topoisomerases IB. PAT requires Mg^2+ ^for relaxation, but not ATP, and high concentration of NaCl cannot substitute for Mg^2+ ^in the reaction, unlike what has been reported for the Vaccinia-encoded homolog. A comparable domain organization and constellation of catalytic residues is suggested by the observation that substitution of the tyrosine nucleophile in the active site leads to a mutant that binds DNA but is unable to relax DNA, and that the catalytic fragment is able to relax supercoiled DNA in the absence of the N-terminal domain.

## Methods

### Cloning and expression of *P. aeruginosa *topoisomerase I

The PAT gene was PCR amplified from the genomic DNA UCBPP-PA14 using forward primer 5'-CGGAACCCACATATGAGCGCAGC-3' and reverse primer 5'-GCACGGCATGGATCCGCACC-3'. NdeI and BamHI restriction sites were incorporated into the oligonucleotide primers, and the fragment containing the *P. aeruginosa *topoisomerase I coding region was cloned into the *E. coli *expression vector pET5a. The identity of the cloned fragment was confirmed by DNA sequencing, and the recombinant plasmid was used to transform *E. coli *strain BL21(DE3)pLysS. The transformants were plated on MDAG media which is an enriched, fully defined non-inducing media; the MDAG media was prepared as described in [30], except the 1000× metals was made according to [31]. Vaccinia topoisomerase IB was purchased from Epicentre (Madison, WI).

### Mutagenesis

The point mutation on *P. aeruginosa *topoisomerase I to replace the tyrosine with phenylalanine at the active site was created using whole plasmid PCR. The sequence of the forward primer is 5'-CGGCAATGCTTCATCCACCCGG-3' and the reverse primer is 5'-GCAGATCGCCACGCTGTTGCC-3'. The template was removed by DpnI digestion and the PCR product was used to transform *E. coli *TOP10 (Invitrogen). The catalytic fragment was created by amplification of the C-terminal region using forward primer 5'-GCGCGAGCATATGGATGCC-3' and reverse primer 5'-CCTTTCGTCTTCAAGAATTCGGATCC-3'. NdeI and BamHI restriction sites were introduced at either end of the PCR product, which was cloned into pET5a. Constructs were confirmed by sequencing.

Plasmids harboring the gene encoding the full length enzyme (PAT), catalytic mutant (PATCAT) or the Y292F mutant were transformed into BL21(DE3)pLysS, cells were grown in LB media at 37°C, 250 rpm, until OD ≈ 0.2 and induced with Isopropyl β-D-1-thiogalactopyranoside (IPTG) at a final concentration of 0.5 mM. After one hour, the cells were harvested by pelleting and over-expression was confirmed by SDS-PAGE followed by Coomassie Brilliant Blue staining. For PAT, Y292F and PATCAT the cells were harvested by centrifugation and the pellets were stored at -80°C. Due to instability of the PAT enzyme, overexpression was also performed at room temperature, but no difference was observed with regard to activity of the purified protein.

### Protein purification

Six 1500 ml cultures of *E. coli *BL21(DE3)pLysS containing plasmid carrying the PAT gene were induced as noted above, cells were pelleted, and the pellet was redissolved in HA buffer (50 mM Tris-HCl, pH 8.0, 1 mM EDTA, 10% glycerol and 0.25 M NaCl, 7.2 mM β-mercaptoethanol, 0.2 mM phenylmethylsulfonyl fluoride (PMSF)) and lysed by sonication; about 20% *P. aeruginosa *topoisomerase I was in the pellet at this step. After removing the insoluble material in the crude lysate by centrifugation at 5,000–6,000 rpm for 15 minutes, the soluble fractions were subjected to 60% ammonium sulfate precipitation. The supernatant remaining after the final precipitation was found to contain 80% *P. aeruginosa *topoisomerase I. The supernatant was dialyzed overnight against HA buffer. The dialyzed supernatant was applied to a phosphocellulose column previously equilibrated to pH 8.0. The elution was performed by applying a step gradient from 0.4–1.5 M NaCl. All steps of purification were performed at 0–4°C. The glycerol concentration in the eluate was increased to 30% and the protein was stored at -20°C.

The pellet from the overexpressed Y292F mutant was dissolved in HA buffer and subjected to 30% ammonium sulfate precipitation. The pellet from the precipitation was redissolved in HA buffer, lysed by sonication and loaded on a heparin agarose column in HA buffer. Protein was eluted by a gradient from 0.4–1.5 M NaCl in HA. The overexpressed PATCAT mutant was similarly subjected to 30% ammonium sulfate precipitation. The supernatant from the precipitation was dialyzed against HA buffer overnight and loaded onto the phosphocellulose column pre-equilibrated in the same buffer. Protein was eluted by step gradient from 0.4–1.5 M NaCl. Protein concentrations were determined by Coomassie-stained SDS-PAGE gels using BSA standards.

### Relaxation assay

Relaxation assay (per 20 μl) was performed with negatively supercoiled pUC18 DNA (≈ 110 ng) unless otherwise stated, and the indicated amount of PAT in topoisomerase buffer (50 mM Tris (pH 8.0), 2.5 mM MgCl_2_, 0.1 mM EDTA) for two hours at 37°C. The reaction also contained a final concentration of 0.24 M NaCl and ≈ 19% glycerol from the protein storage buffer. Reactions were stopped with 5 μl 1(10% SDS):1(30% glycerol) stop buffer. The reactions were loaded on a 1% agarose gel in 0.5× TBE buffer (45 mM Tris-borate (pH 8.3), 1 mM EDTA). The gels were run at 2 V/cm for 16 hours. The gels were stained with ethidium bromide (EtBr) and visualized using an Alpha Innotech digital imaging System.

The positively supercoiled DNA was prepared by first nicking pUC18 with N.BstNBI for an hour, followed by phenol extraction and ethanol precipitation. Nicked pUC18 was then incubated with netropsin and T4 DNA ligase and again phenol extracted and ethanol precipitated to remove the drug, resulting in the production of positively supercoiled DNA. The production of positively supercoiled DNA was confirmed by two dimensional agarose gel electrophoresis, as described [32]. To confirm production of covalently closed DNA on incubation with PAT, the same reactions were electrophoresed both on 1% agarose gels as noted above as well as on a gel containing ethidium bromide.

### Electrophoretic mobility shift assay

Oligonucleotides were purchased from Operon. The top strand oligonucleotides were labeled at the 5' end with T4 polynucleotide kinase (New England Biolabs) and [γ-^32^P] ATP and annealed to the bottom strand. The sequences of the oligonucleotides are given in Table 1.

**Table 1:** The 37 bp DNA constructs.

A.37 bp VacSeq	5'-CGTGTCGATTCCGACGT**CCCTT**GCATTTATCAATTAT-3'
	3'-GCACAGCTAAGGCTGCAGGGAACGTAAATAGTTAATA-5'
B.37 bp loops	5'-CCTAGGCTACACCTACTCTTTGTAAGAATTAAGCTTC-3'
	3'-GGATCCGATGTG**CT**TGAGAAACA**AA**CTTAATTCGAAG-5'
C.37 bp no loops	5'-CCTAGGCTACACCTACTCTTTGTAAGAATTAAGCTTC-3'
	3'-GGATCCGATGTGGATGAGAAACATTCTTAATTCGAAG-5'

**(A) **The sequence of the 37 bp containing the Vaccinia recognition pentameric sequence in bold.**(B) **The sequence of the loop-containing duplex: the sequence of the bottom strand is modified to generate tandem mismatches of identical opposing nucleotides with spacing of 9 bp. The sequence generating the loops is in boldface.**(C) **The sequence of the perfect duplex without loops.

The reaction mixture per 20 μl contained 50 fmol of the labeled DNA substrate which was incubated with the indicated amount of *P. aeruginosa *topoisomerase I or mutant protein in topoisomerase buffer (50 mM Tris (pH 8.0), 2.5 mM MgCl_2_, 0.1 mM EDTA) for one hour at 37°C. The reaction mixture contained 0.67 M NaCl final concentration. The glycerol concentration in each sample was different as noted in the figure legend; this was due to the instability of the protein, which precluded its concentration following elution. Samples were loaded on 8% (w/v) PAGE (37:1 acrylamide/bisacrylamide) gels in 0.5 × TBE and run for 90 minutes. Gels were visualized on a Molecular Dynamics Storm Phosphoimager using software supplied by the manufacturer.

## Results

### Sequence alignment

**Figure 1:**
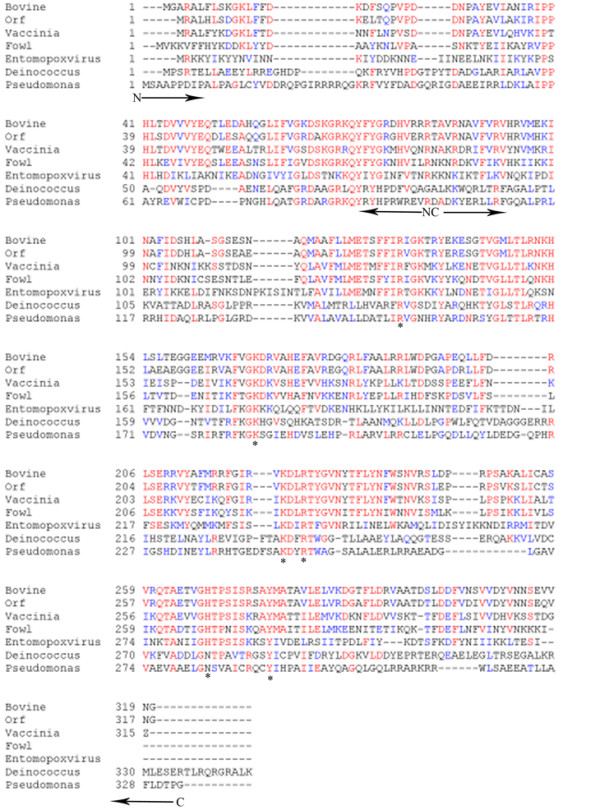
**Sequence alignment of *P. aeruginosa *topoisomerase I with homologous topoisomerases IB**. The amino acid sequences from Entomopoxvirus (MSV130), Bovine Papular Stomatitis virus (BPSVgORF062), Orf virus (ORFVgORF062), Vaccinia virus (H6R, Vaccinia topoisomerase type IB), Fowl Pox virus (FPV143) and *D. radiodurans *topoisomerase IB (DR_0690) are aligned with the *P. aeruginosa *topoisomerase I (PA2G_01347). Sequence gaps are indicated by dashes. The residues in blue and red correspond to identical and conserved residues, respectively. The asterisks in black identify the conserved catalytic residues. The N-and C-terminal domains are demarcated by arrows.

Screening of the NCBI database with the BLAST search engine and PAT as the query yielded several homologs including Vaccinia virus topoisomerase IB. A group of conserved amino acid side chains in the active site coordinate the attack of the tyrosine nucleophile on the scissile phosphodiester. This catalytic pentad, Arg-130, Lys-167, Lys-220, Arg-223 and His-265 as well as Tyr-274 for the Vaccinia virus topoisomerase I, is conserved among the poxvirus enzymes, while the closely related tyrosine recombinases typically contain a histidine in place of the lysine in position 220 of Vaccinia topoisomerase. The two arginines and the histidine interact directly with the scissile phosphodiester and may serve to stabilize a negatively charged transition state [6,33-36]. The homologous residues in *P. aeruginosa *topoisomerase I are Arg-148, Lys-185, Lys-247, Arg-250, Asn-283 and Tyr-292 (indicated with an asterisk in Fig. 1) and in the *D. radiodurans *encoded homolog, Arg-137, Lys-174, Lys-235, Arg-237, Asn-280 and Tyr-289. The His-265 is replaced with Asn-283 in *P. aeruginosa *topoisomerase and Asn-280 in *D. radiodurans *topoisomerase[27]. The *P. aeruginosa *topoisomerase I showed 13% sequence identity with Entomopox virus, 20% with Bovine Papular Stomatitis virus, 23% with Orf virus, 14% with Vaccinia virus IB, 14% with Fowl Pox virus and the *D. radiodurans *topoisomerase aligned particularly well at 36% identity. The C-terminal catalytic domain is structurally conserved, and the catalytic pentad occupies equivalent positions in the structures of the topoisomerases IB and tyrosine recombinases. In contrast, the N-terminal domain is not conserved between topoisomerases and recombinases.

**Figure 2:**
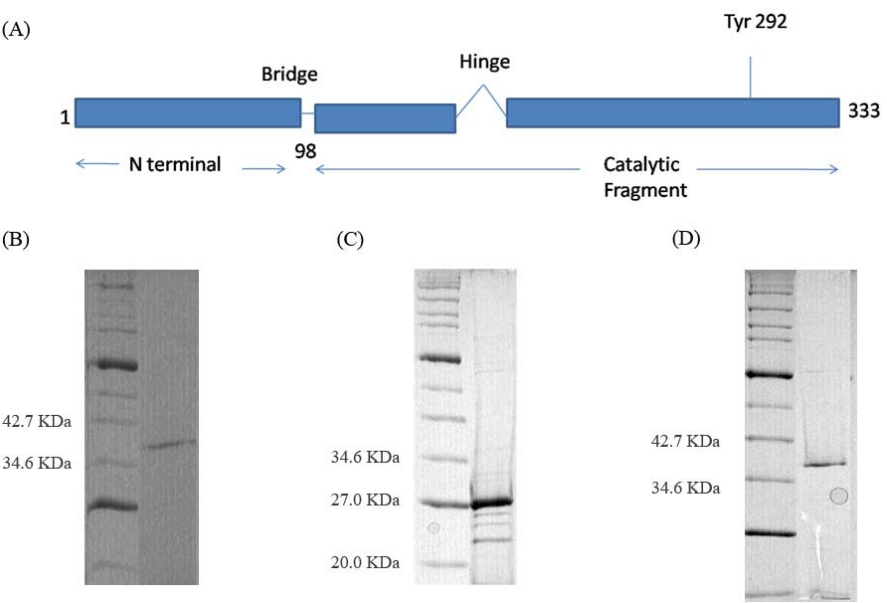
**Domain structure of *P. aeruginosa *topoisomerase I**. **(A) **Schematic representation of the tripartite structure predicted for the 333 amino acid *P. aeruginosa *topoisomerase I is illustrated using the Vaccinia topoisomerase IB as a template. The mutant protein lacking the 98-residue N-terminal domain is the catalytic fragment (PATCAT, residues 99–333). For the active site tyrosine mutant, the active site tyrosine is changed to phenylalanine (referred to as the Y292F mutant protein). **(B) ***P. aeruginosa *topoisomerase I wild type purified. The enzyme fraction was analyzed by SDS-PAGE. In lane 1 is the molecular weight marker, lane 2 shows the PAT. **(C) ***P. aeruginosa *topoisomerase I catalytic fragment purified, lane 1 shows the molecular marker and lane 2 shows the PATCAT **(D) ***P. aeruginosa *topoisomerase tyrosine mutant purified, lane 1 shows the molecular weight marker and lane 2 shows the Y292F mutant protein.

The 333 amino acid *P. aeruginosa *topoisomerase I is predicted to consist of three protease-resistant structural domains demarcated by two protease-sensitive segments referred to as the bridge and the hinge (Fig. 2A) [37]. Residues 1 through 98 constitute the N-terminal domain and residues 99 through 333 are predicted to form the catalytic C-terminal domain. The C-terminal domain, which includes the bridge and the hinge also includes the active site nucleophile (Tyr-292), which is implicated in transesterification. While the N-terminal domains of poxvirus topoisomerases and tyrosine recombinases are divergent, this domain is conserved between poxvirus and bacterial topoisomerases. To establish whether the *P. aeruginosa*-encoded homolog is functional, we cloned the gene and characterized the purified protein. Based on sequence features, we also created two mutants of PAT; one is the truncated C-terminal domain consisting of residues 99–333 (PATCAT) and the other is the active site mutant where the active site tyrosine nucleophile is changed to phenylalanine, referred to here as Y292F. Initial expression analyses of PAT revealed that the protein is highly unstable and loses activity upon storage. For this reason, we performed a relaxation assay each time before using the PAT for other experiments. Under typical growth conditions, the protein was also toxic, and showed poor expression when expressed using BL21(DE3)pLysS; therefore, the transformants were grown on non-inducing media containing low concentrations of glucose and amino acids. Glucose and amino acids prevent induction by lactose during log phase growth, and metabolic balancing of pH allowed development of reliable non-inducing media. Expression strains grown on non-inducing media retain plasmids and remain fully viable for months in the refrigerator. While comparable DNA binding sites for PAT and PATCAT may be suggested by the observation that both proteins interact strongly with phosphocellulose, the Y292F mutant does not bind to the phosphocellulose and was instead purified using heparin agarose chromatography. The purified wild type and mutant proteins are shown in Fig. 2B.

### DNA relaxation activity of *P. aeruginosa *topoisomerase I

**Figure 3:**
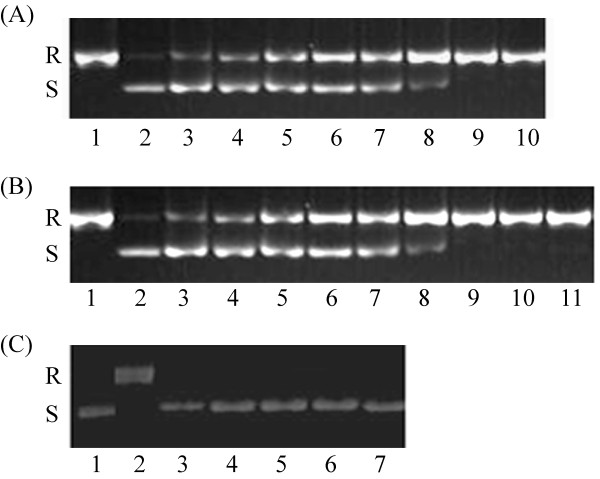
**(A) Kinetics of DNA relaxation by PAT**. Lane 1 shows relaxed DNA. Supercoiled pUC18 (110 ng) was relaxed with 9 ng PAT in topoisomerase buffer. Lanes 2 through 10 contain 110 ng of DNA and 9 ng PAT. Reactions were stopped after 0.5, 1, 2, 3, 4, 5, 10, 15 and 20 minutes. The relaxed DNA (R) and supercoiled DNA (S) are identified on the left.**(B) **Relaxation of supercoiled pUC18 in the presence of PATCAT. Lane 1 shows the relaxed DNA. The supercoiled pUC18 (110 ng) was relaxed with 9 ng PAT i. Lanes 2 through 11 contain 110 ng DNA and 808.2 ng PATCAT. Reactions were stopped after 0.5, 1, 2, 3, 4, 5, 10, 15, 20, and 25 minutes in lanes 2 to 11 respectively. The relaxed and supercoiled DNA are identified by (R) and (S) on the left. **(C) **The Y292F mutant is not able to relax supercoiled pUC18. The relaxed and supercoiled DNA are identified by (R) and (S) on the left. In lane 1, is shown the negative control containing supercoiled pUC18 and lane 2 is showing the relaxed DNA (supercoiled pUC18 (110 ng) relaxed with PAT (9 ng)). Lanes 3 through 7 contain 110 ng DNA and 241 ng Y292F. Reactions were stopped after 5, 10, 15, 20 and 25 minutes, respectively.

**Figure 4:**
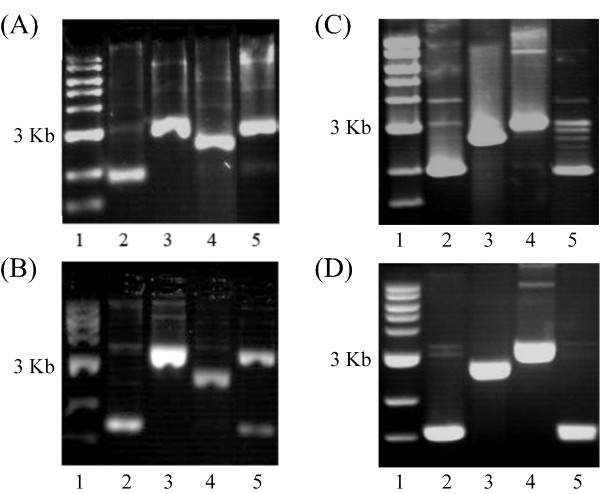
**PAT produces covalently closed, relaxed DNA**. **(A) **Lane 1 contains the 1 Kb ladder, lane 2 contains 120 ng of supercoiled DNA, lane 3 contains 130 ng nicked DNA, lane 4 contains 140 ng linear DNA and lane 5 contains 140 ng supercoiled pUC18 and 63 ng protein in topoisomerase buffer with MgCl_2_. The reaction mixtures were run on 1% agarose gel. **(B) **Lane 1 contains the 1 Kb ladder, lane 2 contains 120 ng supercoiled DNA, lane 3 contains 130 ng nicked DNA, lane 4 contains 140 ng linear DNA, lane 5 contains 140 ng supercoiled pUC18 and 63 ng protein in topoisomerase buffer with MgCl_2_. The reaction mixtures were run on a 1% agarose gel with 2 μg/ml ethidium bromide. Samples in panels A and B represent the same reaction, half of which is loaded on each gel. For reactions in lanes 5, quantitation of relative amounts of supercoiled DNA shows that <8% of total DNA remains supercoiled after incubation with topoisomerase (panel (A)), while ~35% of total DNA is supercoiled after electrophoresis in the presence of ethidium bromide (panel (B)), indicating that ~30% of total DNA is covalently closed following reaction with PAT. Panels **(C)** and **(D)** are equivalent to (A) and (B) with lanes 2–5 containing supercoiled, linear, nicked, and relaxed DNA, respectively; DNA in lanes 5 is relaxed with Vaccinia topoisomerase IB.

DNA relaxation activity of the *P. aeruginosa *topoisomerase I enzyme was assayed by incubation with supercoiled plasmid pUC18. Reaction mixtures containing freshly prepared *P. aeruginosa *topoisomerase I and supercoiled pUC18 were incubated for different times and the reactions were terminated with SDS. The full-length enzyme (9.2 ng) starts to relax 110 ng supercoiled pUC18 after 0.5 minutes and all the DNA is relaxed in 15 minutes; in comparison, the less efficient PATCAT (808.2 ng) starts to relax 110 ng of supercoiled pUC18 after 0.5 min and the DNA is completely relaxed in 15 minutes (Fig. 3A and 3B). For Vaccinia topoisomerase, 2.7 ng enzyme relaxes 300 ng supercoiled pUC18 in 0.25 min in the presence of Mg^2+ ^and in the absence of Mg^2+ ^it relaxes DNA in 2 minutes [38]. The *D. radiodurans *topoisomerase IB is also not as efficient as the Vaccinia homolog, about 350 ng enzyme relaxes 300 ng of supercoiled pUC18 in 5 minutes [27]. Consistent with its role in the transesterification reaction, the Y292F mutant is unable to relax supercoiled DNA (Fig. 3C). To determine if the product of PAT-mediated DNA relaxation is covalently closed, reactions were divided in two parts and one part electrophoresed on gels containing ethidium bromide, under which conditions only covalently closed DNA will become supercoiled. As seen in Fig. 4, PAT does produce covalently closed product (compare lanes 5). Quantitation of relative amounts of relaxed and supercoiled DNA (lanes 5) show that the majority of the supercoiled DNA seen in the presence of ethidium bromide indeed corresponds to covalently closed relaxed DNA that has become supercoiled in the presence of ethidium bromide, and not residual supercoiled DNA substrate. Note also that the mobility of DNA that is supercoiled in the presence of ethidium bromide is the same as that of the original substrate DNA, as evidenced by the observation that DNA relaxed by Vaccinia topoisomerase IB likewise migrates to the same position in the ethidium bromide-containing gel as the supercoiled substrate DNA (panels C-D). We also note that the fraction of nicked DNA appears to increase as the protein preparation ages, reflecting instability of the enzyme.

**Figure 5:**
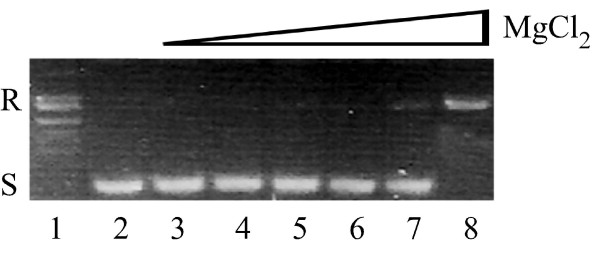
**MgCl_2 _is required for DNA relaxation**. Lane 1 shows relaxed DNA (R) containing 110 ng pUC18 DNA and 9 ng PAT in topoisomerase buffer with 2.5 mM MgCl_2 _and lane 2 shows supercoiled (S) DNA without PAT. Reaction mixtures containing 110 ng of supercoiled pUC18, 9 ng of PAT and increasing concentration of MgCl_2_, 0, 0.00025, 0.0025, 0.025, 0.25, 2.5 mM in lanes 3–8, respectively.

**Figure 6:**
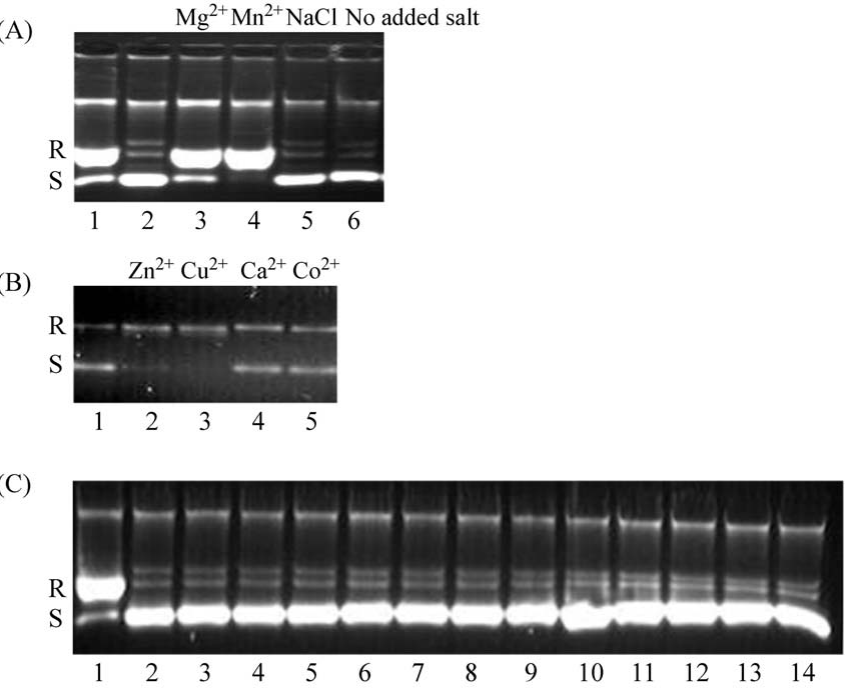
**(A) Relaxation of supercoiled DNA in the presence of different cations**. Reactions contained 200 ng of supercoiled pUC18 and the indicated cation at 2.5 mM final concentration in topoisomerase buffer without MgCl_2 _and 0.24 M NaCl. Lane 1: relaxed DNA (R); relaxation of 200 ng supercoiled pUC18 was performed with 9 ng of PAT in topoisomerase buffer with MgCl_2 _for 2 hours at 37°C. Lane 2 contains 200 ng supercoiled pUC18 in the topoisomerase buffer indicated by (S) on the left hand side. Lanes 3 through 6 contain 9 ng of PAT. **(B) **Reactions contain 75 ng of supercoiled pUC18, 9 ng PAT, indicated cation at 2.5 mM concentration. The relaxed DNA (R) and supercoiled pUC18 (S) are identified on the left and were obtained as described in (A). Control reactions with metals only and no enzyme reveal no nicking activity (data not shown). **(C**) Effect of NaCl on topoisomerase activity. Reaction mixtures contained 110 ng of supercoiled pUC18 DNA, 2.5 mM EDTA, 9 ng PAT, and increasing concentration of NaCl 265 mM, 290 mM, 315 mM, 340 mM, 365 mM, 390 mM, 415 mM, 440 mM, 465 mM, 490 mM, 540 mM and 590 mM in lanes 3 through 14 respectively in topoisomerase buffer without MgCl_2_. Lane 1 shows relaxed DNA and lane 2 is the supercoiled pUC18 DNA. The relaxed DNA (R) and supercoiled pUC18 (S) are identified on the left and were obtained as described in (A). Gels in panels (A) and (C) were electrophoresed at a higher voltage, reducing the separation between relaxed and supercoiled species.

Surprisingly, PAT is unable to relax DNA in the absence of MgCl_2 _(Fig. 5). When titrating MgCl_2 _from 0 to 2.5 mM, *P. aeruginosa *topoisomerase I (202 fmole) was seen to relax supercoiled pUC18 DNA at 2.5 mM MgCl_2_. This is in contrast to Vaccinia topoisomerase I which was reported not to require Mg^2+ ^for activity although its activity is stimulated in its presence [14]. DNA relaxation by *P. aeruginosa *topoisomerase I with different salts in the reaction mixtures showed that the enzyme requires divalent cations for relaxation, and that high concentration of NaCl cannot substitute for MgCl_2 _in the reaction mixture. A more detailed examination of the cation specificity is shown in Fig. 6A–B. MgCl_2_, MnCl_2_, CuCl_2 _and ZnCl_2 _had a stimulatory effect on the relaxation activity. For Vaccinia topoisomerase I, higher concentration of monovalent salt can substitute for MgCl_2_. By contrast, high concentration of NaCl cannot substitute for MgCl_2 _in the reaction catalyzed by PAT (Fig. 6C). Calcium and cobalt cannot substitute for MgCl_2 _at relaxation of supercoiled DNA when compared with zinc, copper, magnesium and manganese (control reactions performed with metals only and no enzyme reveal no nicking activity; data not shown). The *P. aeruginosa *topoisomerase I differs from Vaccinia virus topoisomerase IB in the sense that Vaccinia virus topoisomerase IB does not relax DNA in the presence of ZnCl_2 _and for *P. aeruginosa *topoisomerase I, ZnCl_2 _is able to substitute for MgCl_2 _in the relaxation reaction (Fig. 6A, B) [14]. PATCAT shares similar requirements for divalent cations as PAT (data not shown).

Vaccinia virus topoisomerase IB requires a monovalent salt for relaxation activity and in the presence of EDTA the activity was optimal between 100 and 250 mM NaCl [14]. This was not true for *P. aeruginosa *topoisomerase I which fails to relax supercoiled DNA under comparable conditions (Fig. 6C). High [NaCl] (1 M), however, was inhibitory for enzyme activity (data not shown).

**Figure 7:**
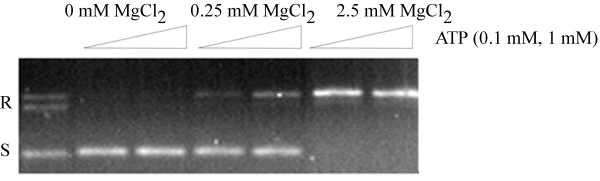
**PAT does not require ATP for relaxation of supercoiled DNA**. All reactions contain 110 ng of supercoiled pUC18 and 9 ng of PAT. R indicates relaxed DNA and S indicates supercoiled pUC18 in lane 1. Lane 2 contains 0.1 mM ATP and 0 mM MgCl_2_, lane 3 contains 1 mM ATP and 0 mM MgCl_2_, lane 4 contains 0.1 mM ATP and 0.25 mM MgCl_2_, lane 5 contains 1 mM ATP and 0.25 mM MgCl_2_, lane 6 contains 0.1 mM ATP and 2.5 mM MgCl_2_, lane 7 contains 1 mM ATP and 2.5 mM MgCl_2_.

**Figure 8:**
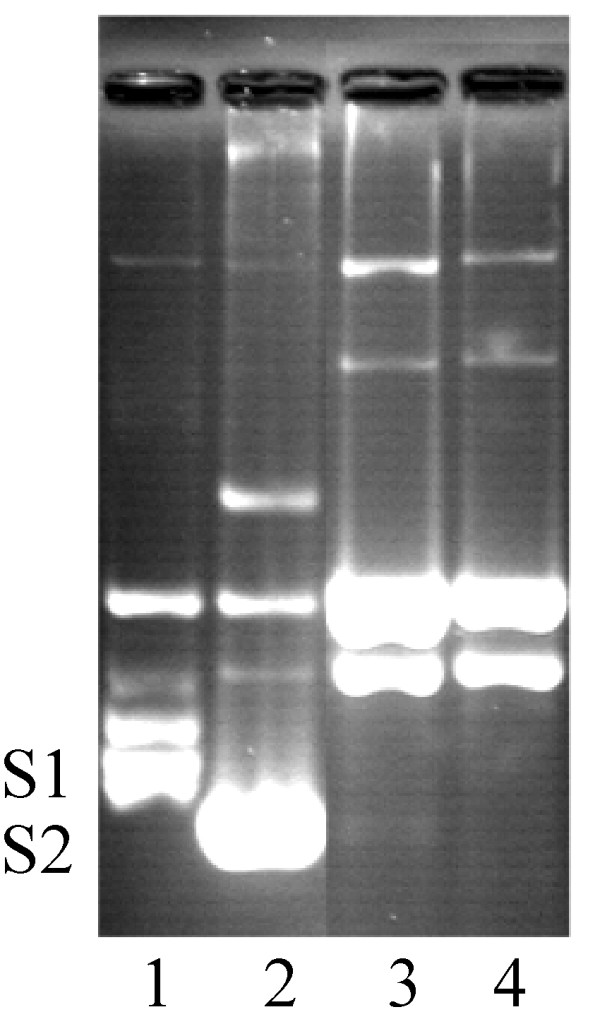
**PAT relaxes positively supercoiled DNA as well as negatively supercoiled DNA**. In lane 1 is positively supercoiled pUC18 (S1), lane 2 contains negatively supercoiled pUC18 (S2), lane 3 contains positively supercoiled DNA relaxed, lane 4 contains negatively supercoiled DNA relaxed. Lanes 3 and 4 contain 110 ng of supercoiled DNA, 9 ng PAT in the topoisomerase buffer with 2.5 mM MgCl_2_.

The type II topoisomerases utilize ATP for activity, while the Type IA and IB topoisomerases do not require ATP to relax DNA. Type I topoisomerases can thus be differentiated from type II enzymes by either the requirement of ATP or by the fact that the type I enzymes bring a change in ± 1 in the linking number and type II change the linking number by ± 2 [39]. As shown in Fig. 7, PAT does not require ATP for relaxation, nor does ATP inhibit activity. Eukaryotic type I topoisomerases differ from the prokaryotic type I topoisomerase in the ability to relax positively supercoiled DNA [40]. Indeed, Vaccinia topoisomerase IB was reported to relax positively supercoiled DNA more efficiently [41]. Notably, PAT is able to relax positively supercoiled DNA (Fig. 8). We also note that the observed activity is unlikely to derive from contaminating *E. coli *topoisomerases; type II enzymes would be sensitive to fluoroquinolone antibiotics (and yield a linear, not a nicked intermediate), and the activity observed here is insensitive to ciprofloxacin (data not shown). While contaminating Type IA activity may not be readily addressed using inhibitors, such activity would be expected to result in a population of topoisomers with different linking numbers (e.g., [42]). Contaminating Tyr recombinases are not likely either due to the observed requirement for divalent metal ions for activity, indeed, contaminating XerC/D recombinases can be ruled out, as plasmid pUC18 used for relaxation assays has no site for these site-specific enzymes. As we consistently observe the generation of covalently closed and nicked, relaxed species, we conclude that the observed activity (which is absent in the preparations of Y292F and significantly reduced for PATCAT) is due to PAT, and that this enzyme relaxes supercoiled DNA to completion following DNA strand incision.

### Sequence preference of PAT

**Figure 9:**
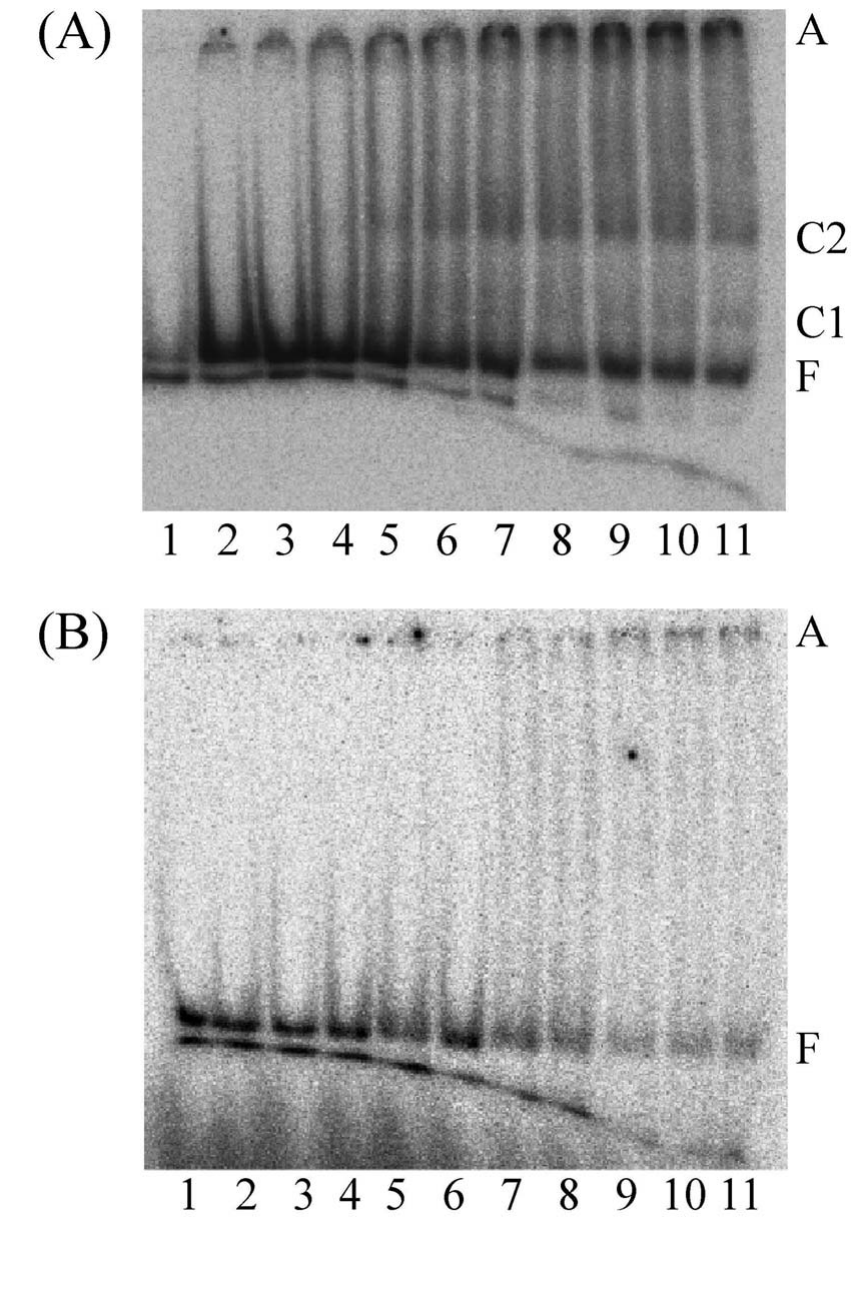
**Binding of PAT to 37 bp DNA with and without CCCTT-containing sequence**. **(A) **PAT 0, 20, 40, 68, 136, 200, 268, 336, 400, 468, 712 fmol respectively in lanes 1–11 in topoisomerase buffer with MgCl_2_. The glycerol concentration in lanes 1 through 11 varies from 8.34% to 24.5%. Free DNA (F), complex (C) and aggregates (A) are indicated on the right.**(B) **Binding of PAT to 37 bp perfect duplex DNA; PAT 0, 20, 40, 68, 136, 200, 268, 336, 400, 468, 712 fmol respectively in lanes 1–11 in topoisomerase buffer without MgCl_2_. The glycerol concentration varies from 8.34% to 24.5% in lanes 1 through 11. Free DNA (F) and aggregates (A) are indicated on the right.

**Figure 10:**
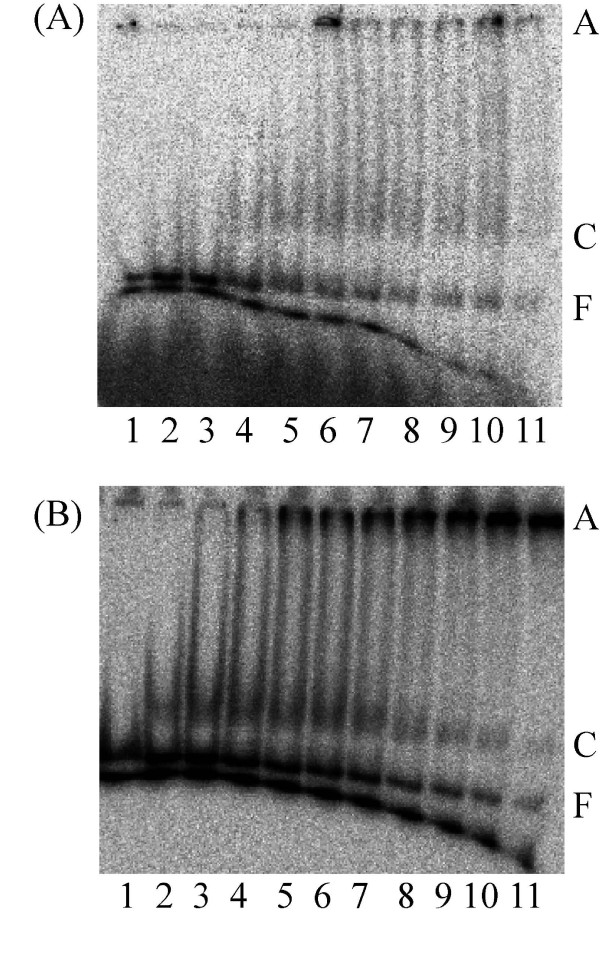
**Binding of PAT and Y292F to 37 bp looped DNA**. **(A) **PAT 0, 20, 40, 68, 136, 200, 268, 336, 400, 468, 712 fmol respectively in lanes 1–11 in topoisomerase buffer with MgCl_2 _and 37 bp looped DNA. The glycerol concentration in lanes 1 through 11 varies from 8.34% to 24.5%. Free DNA (F), complex (C) and aggregates (A) are indicated on the left. **(B) **Binding of Y292F to 37 bp looped DNA. Y292F 0, 0.53, 1.06, 1.8, 3.6, 5.3, 7.1, 8.9, 10.6, 12.4, 18.8 pmol respectively in lanes 1–11 in topoisomerase buffer with MgCl_2 _and 37 bp looped DNA. The glycerol concentration in lanes 1 through 11 varies from 8.3% to 32.26%. Free DNA (F), complex (C) and aggregates (A) are indicated on the right.

Vaccinia topoisomerase I is specific for cleavage of the sequence (C/T)CCTT↓ in the scissile strand. To evaluate the sequence preference of PAT, we therefore first compared complex formation with 37 bp DNA containing the Vaccinia topoisomerase recognition sequence, a 37 bp DNA of average G+C content, or the equivalent 37 bp DNA in which two 4 nucleotide loops were introduced (Table 1). In addition, a previously published shorter duplex used to monitor Vaccinia topoisomerase cleavage kinetics was used; PAT does not yield a complex with this 18/24 duplex DNA used by the Vaccinia virus topoisomerase IB (data not shown) perhaps because it is too short for stable complex formation. However, PAT does bind the 37 bp DNA containing the pentameric Vaccinia sequence and forms two complexes as seen in Fig. 9A. Some preference for the CCCTT-containing DNA is suggested by the observation that PAT does not form a discrete complex with the 37 bp perfect duplex (Fig. 9B), but it does interact more stably with the corresponding looped DNA (Fig. 10A). The Y292F mutant also binds DNA (37 bp looped DNA Fig. 10B) but does not relax DNA. Regardless of DNA substrate, the presence of MgCl_2 _in the reaction has no effect on complex formation (data not shown).

The PAT forms two distinct complexes with 37 bp CCCTT-containing DNA. It might be that the enzyme binds to the DNA at different sites, thus giving rise to more than one complex of distinct mobility. Maybe one PAT molecule binds to CCCTT, which in turn causes a second PAT to bind DNA, resulting in complexes that are stuck in the well. The preferred binding to 37 bp DNA containing the Vaccinia topoisomerase site suggests that PAT recognizes certain sequence elements. While all poxvirus topoisomerases favor cleavage at (C/T)CCTT sequences [15], surrounding sequences also effects DNA cleavage [43]; for example, only four of the eight CCCTT sequences in pUC19 are cleaved by MCV topoisomerase [26]. However, the *D. radiodurans *encoded homolog was reported not to share the preference for cleavage at (C/T)CCTT sites as evidenced by the failure to detect covalent adduct with CCCTT-containing duplex DNA [27]. Similarly, we have been unable to capture this covalent adduct using the 37 bp DNA (data not shown), reflecting either a lack of preference for cleavage at this site or a religation reaction that is too rapid to allow detection. For poxvirus-encoded enzymes, such covalent adducts have been readily detected; in contrast, for other topoisomerases, the covalent intermediate is quite short-lived and generally cannot be captured in absence of specific inhibitors or without the use of suicide substrates in which the non-covalently held DNA dissociates to prevent religation. That both bacterial topoisomerases fail to yield detectable covalent intermediate suggest a mechanistic difference from poxvirus-encoded homologs, either in terms of substrate recognition or religation kinetics.

## Discussion

Few bacterial species encode a type IB topoisomerase with similarity to the poxvirus-encoded enzymes, and *P. aeruginosa *topoisomerase I is only the second bacterial homolog to be characterized and shown to be functional *in vitro*. The structural gene for the *P. aeruginosa *topoisomerase I when induced to express in *E. coli *results in the appearance of the expected Mr 38 KDa polypeptide which is responsible for the relaxation activity. While PAT relaxes both positively and negatively supercoiled DNA in absence of ATP and appears to retain the domain organization and constellation of catalytic residues characteristic of the poxvirus-encoded enzymes, PAT does exhibit a unique requirement for divalent cations for catalytic activity.

### Effect of salt on relaxation activity

Analysis of the purified enzyme showed that while PAT does not require any divalent cation for DNA binding, it does require divalent cations for relaxation of supercoiled DNA, which suggests that either the divalent cations are required for cleavage complex formation or for religation of DNA after removal of the torsional strain. The divalent cation could also play a role in site-specific binding, including the enzyme's ability to discern the topological state of the nucleic acid substrate. The poxvirus topoisomerases were reported not to require Mg^2+ ^for binding and cleavage, however, the Vaccinia enzyme activity is stimulated 10- to 20-fold by divalent cations.

### Domain organization of PAT

The catalytic fragment (235 amino acids) of *P. aeruginosa *topoisomerase I lacking the amino terminal domain is able to relax DNA, albeit less efficiently than the full-length enzyme. Similarly, the Vaccinia topoisomerase catalytic domain is less efficient than the full length enzyme, a phenomenon ascribed to lower affinity binding to the CCCTT target sequence and a reduced rate of DNA cleavage [38]. The structure of poxvirus topoisomerase IB in complex with its cognate DNA site confirms the contribution of the N-terminal domain to site-specific binding [44]. A β-strand from the N-terminal domain makes contacts to several bases within the CCCTT sequence; these residues (Tyr-70, Tyr-72 and Gln-69 of poxvirus topoisomerase) are all conserved in the *P. aeruginosa*-encoded homolog. Thus, removal of the N-terminal domain of PAT may likewise attenuate any sequence-preferences exhibited by the full-length enzyme. Failure to engage the cognate site adequately may in turn result in suboptimal positioning of catalytic residues. Consistent with this interpretation, mutation of Tyr-70, Tyr-72 and Gln-69 of poxvirus topoisomerase results in defects in relaxation activity [44].

**Figure 11:**
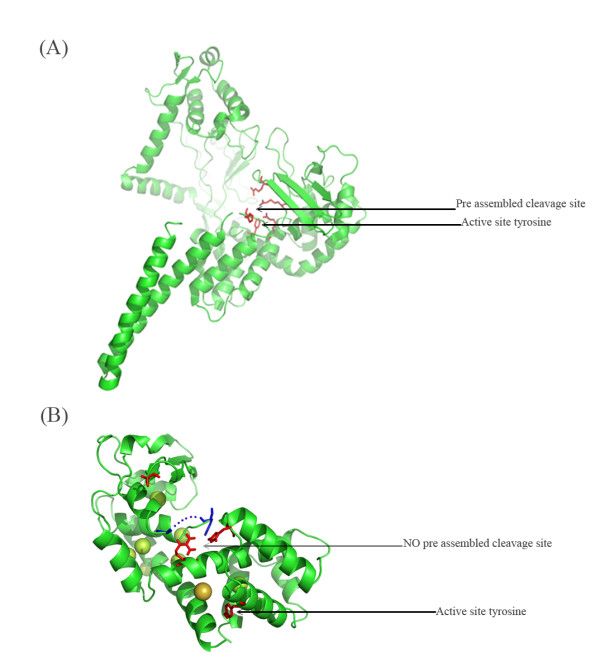
**In the figure is shown the cleavage site assembly for the human topoisomerase (PDB IK4T) (panel A) and Vaccinia topoisomerase IB (PDB IA4I) (panel B)**. The human topoisomerase IB has a preassembled precleavage site but in the Vaccinia enzyme, the catalytic tyrosine is located away from the cleavage site and is reoriented after DNA binding.

The human topoisomerase (Fig. 11A) and the *Deinococcus *topoisomerase have a preassembled cleavage site as opposed to the Vaccinia virus topoisomerase in which the active site is not preassembled prior to DNA binding (Fig. 11B) [8]. Comparison of the structures shows that three of the Vaccinia topoisomerase catalytic residues, Arg-130, Lys-167 and Tyr-274 are not in the correct position for transesterification to occur [36,45]. A precleavage conformational change in the catalytic domain would be necessary to orient Tyr-274 to attack the scissile phosphate in the DNA backbone [2,39,46], a conformational change triggered by specific interaction with either strand of the CCCTT target sequence [44]. Based on the sequence conservation between the bacterial enzymes and the properties of the Y292F mutant, we speculate that PAT may likewise have a preassembled cleavage site. While the PAT and PATCAT were both purified by chromatography on phosphocellulose, the Y292F mutant did not bind to phosphocellulose and was purified on the heparin column. If Y292F were far removed from the active site, its replacement with Phe would be unlikely to alter features of the active site. Therefore, we hypothesize that by changing the tyrosine at the active site, the cleavage site was altered, resulting in attenuated interaction with the phosphocellulose column. That this alteration is likely subtle is indicated by the observation that DNA-binding properties of wild type PAT and the Y292F mutant are comparable, including preferred binding to CCCTT-containing and looped DNA.

### DNA binding by PAT

PAT binds to the 37 bp CCCTT-containing sequence as opposed to little binding and no distinct complexes with 37 bp perfect duplex without this pentameric sequence. Both N-and C-terminal domains of the poxviral enzymes contribute to sequence-specific binding, and several residues involved in direct contacts are conserved, notably Tyr-70 and Tyr-72 that contact the central CCT of the CCCTT recognition sequence and Arg-80 and Lys-167 that along with Tyr-72 contact the TT at the scissile phosphate; Arg-80 is not conserved in the *D. radiodurans*-encoded enzyme. However, Lys-133 which contacts the G opposite the less stringently conserved 5'-C and contributes to positioning Arg-130 appropriately at the active site is not conserved in the bacterial homologs, and neither is Asp-168 that makes a water-mediated contact to A at the scissile phosphate [44]. It is therefore conceivable that the bacterial enzymes exhibit only a modest preference for the sequence stringently preferred by the poxviral enzyme. PAT also binds 37 bp looped DNA; this could reflect a binding mode similar to that of eukaryotic topoisomerases which exhibit preferential binding to curved DNA [47,48].

Human topoisomerase IB has an extensive network of ionic contacts to the DNA [2]. These contacts are proposed to restrict rotation of the non-covalently held DNA, resulting in a "controlled rotation" event in which a single superhelical turn is released per cleavage and religation cycle. By contrast, Vaccinia topoisomerase IB releases an average of five superhelical turns prior to religation, leading to the suggestion that non-covalently held DNA rotates freely [49]. Considering the lack of conservation of several residues seen to contact DNA in the poxviral enzymes, it is conceivable that *P. aeruginosa *topoisomerase releases superhelical turns in a completely unhindered rotation event, the end result being complete DNA relaxation before religation can occur. That a significant amount of nicked DNA is detected following DNA relaxation by PAT may be a consequence of enzyme instability, as such deleterious nicked products are unlikely to accumulate *in vivo*. Alternatively, it is possible that PAT acts in concert with other factors *in vivo*, resulting in suboptimal activity *in vitro*; this phenomenon may indeed be related to the observed complete relaxation of DNA if associated factors serve to restrain non-covalently held DNA, thus aiding the religation process. For PAT to exhibit recombinase activity *in vivo *is less likely, however, considering the conservation of the N-terminal domain, which is divergent for the otherwise closely related Tyr recombinases but conserved among poxviral and bacterial topoisomerase homologs.

The reactions catalyzed by PAT proceed via an intermediate in which there is a covalent linkage between the DNA and the enzyme. Such complexes can generally be captured on denaturing gels, however, this complex has not been captured for PAT. There could be several reasons for this. PAT may exhibit a high degree of sequence preference in selection of the cleavage site, a sequence preference only partly emulated by the CCCTT sequence or its context. Secondly, PAT might be recognizing not only sequences but also certain structures in the DNA, perhaps structures promoted by DNA supercoiling. Thirdly, the reaction may be so fast that a suicide substrate needs to be defined in order to capture the cleavage complex. Based on the partial conservation of residues involved in sequence specific DNA interactions we propose that bacterial type IB topoisomerases exhibit a relaxed sequence preference, reflected in failure to capture a cleavage complex with CCCTT-containing DNA and a reduced relaxation activity compared to the poxviral enzymes.

## Conclusion

*P. aeruginosa *encodes a type I topoisomerase with sequence and biochemical characteristics similar to those of the Vaccinia virus topoisomerase IB. Its domain organization resembles that of Vaccinia topoisomerase IB in that residues in the active site are conserved, and a truncated variant lacking the N-terminal domain retains catalytic activity, albeit with lower efficiency compared to the full-length enzyme. In contrast to the Vaccinia-encoded homolog, the *P. aeruginosa*-encoded enzyme requires divalent cations for catalytic activity, relaxes DNA to completion, and shows an only modest preference for the CCCTT pentameric sequence required for transesterification by Vaccinia topoisomerase IB. Analysis of the sequence and structural information suggests that the bacterial topoisomerases IB exhibit reduced activity compared to the poxviral enzymes due to lack of conservation of residues required for optimal positioning of catalytic residues.

## Abbreviations

PAT: *Pseudomonas aeruginosa *topoisomerase I wild type; PATCAT: *Pseudomonas aeruginosa *topoisomerase I catalytic domain; Y292F: Tyrosine mutant of the *Pseudomonas aeruginosa *topoisomerase I; EMSA: Electrophoretic Mobility Shift Assay.

## Authors' contributions

TJ and AG conceived and designed experiments. TJ and BR performed experiments. TJ and AG analyzed data and wrote the paper. All authors read and approved the final manuscript.
